# PEDV enters cells through clathrin-, caveolae-, and lipid raft-mediated endocytosis and traffics via the endo-/lysosome pathway

**DOI:** 10.1186/s13567-020-0739-7

**Published:** 2020-02-10

**Authors:** Xiaona Wei, Gaoli She, Tingting Wu, Chunyi Xue, Yongchang Cao

**Affiliations:** State Key Laboratory of Biocontrol, School of Life Sciences, Sun Yat-sen University, Guangzhou, 510006 People’s Republic of China

## Abstract

With the emergence of highly pathogenic variant strains, porcine epidemic diarrhea virus (PEDV) has led to significant economic loss in the global swine industry. Many studies have described how coronaviruses enter cells, but information on PEDV invasion strategies remains insufficient. Given that the differences in gene sequences and pathogenicity between classical and mutant strains of PEDV may lead to diverse invasion mechanisms, this study focused on the cellular entry pathways and cellular transport of the PEDV GI and GII subtype strains in Vero cells and IPEC-J2 cells. We first characterized the kinetics of PEDV entry into cells and found that the highest invasion rate of PEDV was approximately 33% in the IPEC-J2 cells and approximately 100% in the Vero cells. To clarify the specific endocytic pathways, systematic research methods were used and showed that PEDV enters cells via the clathrin- and caveolae-mediated endocytosis pathways, in which dynamin II, clathrin heavy chain, Eps15, cholesterol, and caveolin-1 were indispensably involved. In addition, lipid raft extraction assay showed that PEDV can also enter cells through lipid raft-mediated endocytosis. To investigate the trafficking of internalized PEDV, we found that PEDV entry into cells relied on low pH and internalized virions reached lysosomes through the early endosome–late endosome–lysosome pathway. The results concretely revealed the entry mechanisms of PEDV and provided an insightful theoretical basis for the further understanding of PEDV pathogenesis and guidance for new targets of antiviral drugs.

## Introduction

As a type of alphacoronavirus, porcine epidemic diarrhea virus (PEDV) has caused enormous economic loss to the global pork industry, especially after the emergence of highly pathogenic PEDV variant strains in 2010. PEDV was first reported in 1971 in the UK [1], and afterward was also discovered in Europe and Asia [2–5]. Although PEDV has persisted in Asian swine-producing countries, it does not attract enough global attention. After October 2010, severe PED outbreaks occurred even in Chinese pig farms that were already vaccinated with CV777-inactivated or live-attenuated vaccines [2, 6–8]. In 2013, the first PEDV outbreak occurred in the US and rapidly spread across the entire country [9]. Molecular epidemiological results show the genetic differences between classical (GI subtype) and new PEDV variant strains (GII subtype) [2, 3, 5, 6, 8, 9]. PEDV is presently recognized worldwide due to dramatic changes observed in its epidemic character, pathogenic properties, and gene drift [10]. Studies focused on its pathogenesis [11, 12], immune evasion [13] and developing effective vaccines [14, 15] are progressing.

A virus is a non-cellular life form that must rely on cells to complete its life cycle. The first step in virus infection is successful entry into cells. Most enveloped viruses enter cells through cellular endocytosis [16, 17]. The endocytic pathways utilized by viruses vary, including clathrin-mediated endocytosis (CME), caveolae-mediated endocytosis, lipid raft-mediated endocytosis, and macropinocytosis, among others. CME is the most classical and well-known endocytic pathway utilized by viruses. After binding to cell surface receptors, the virus is packaged by clathrin-coated pits (CCPs) and transported to clathrin-coated vesicles (CCVs), in which virus particles as the “cargo” will be transported to the early endosomes [18, 19]. Caveolae is a plasma-specific invagination structure with a diameter of 50–100 nm. When viral particles interact with receptors, caveolae coated with caveolin-1 invaginates and pinches off plasma membrane, then the caveolae vesicles mature into caveosomes and deliver “cargoes” to early endosomes [20, 21]. Lipid raft are plasma membrane microdomains enriched in sphingolipids and cholesterol that participate in the lateral organization of the cell surface. Raft-mediated endocytosis is the process of internalization of ligands and receptors by these domains [22].

The mechanisms of some coronaviruses entry into cells have already been studied, such as severe acute respiratory syndrome coronavirus (SARS-CoV), murine hepatitis virus (MHV), and human coronavirus (HCoVs). Entry of SARS-CoV into HepG2 and COS7 cells is clathrin-dependent while entry into Vero E6 cells is clathrin- and caveolae-independent [23, 24], but the lipid raft plays an important role in the process [25]. MHV entry into cells needs clathrin [26–28], the same as HCoV-NL63 [29]. For HCoV-229E, caveolae-mediated endocytosis is utilized to enter human fibroblast cells [30]. SARS-CoV and MHV-CoV can induce continuous micropinocytosis, but this occurs in the later phase during infection and is not associated with virus entry [31]. Coronaviruses enter host cells via various endocytic pathways after viral spike glycoprotein (S) interacts with receptors and then initiates the endocytic process. Internalized viruses are trafficked like cargoes to membrane fusion sites through specific transport routes. Different CoVs have varying fusion sites [32]. The fusion site of the middle east respiratory syndrome coronavirus (MERS-CoV) takes place in the early endosome, while MHV and the feline infectious peritonitis virus (FIPV) are transported to the lysosome to fuse.

Although there have been many studies on the invasion mechanism of CoV, the invasion strategy of PEDV has not yet been fully elucidated. In 2014, Park et al. [33] revealed that PEDV entry followed clathrin-mediated endocytosis and was dependent on a low pH for successful entry into cells. In their research, one PEDV strain was studied in Vero cells in the presence of trypsin and only the chemical inhibitors and confocal method were used to reveal the PEDV entry. However, there are still important questions to address. Considering that CoVs take advantage of different pathways to enter cells, whether different subtypes of PEDV invade cells by different ways and whether PEDV enter different types of cells through different ways remains to be determined. To concretely clarify the entry and transportation routes of PEDV, we used Vero and IPEC-J2 cells as models for PEDV entry and chose CV777-like strain GDS09 and highly pathogenic variant strain GDS01 to compare the invasion strategies of different PEDV subtypes. Our results will advance the understanding of the pathogenesis and immune evasion of PEDV.

## Materials and methods

### Cells, viruses, reagents, antibodies and plasmids

Vero cells were cultured in Dulbecco’s Modified Eagle Medium (DMEM) supplemented with 10% fetal bovine serum (FBS, Gibco) and antibiotics (100 U/mL penicillin and 100 μg/mL streptomycin). IPEC-J2 cells were grown in DMEM/nutrient mixture F-12 (DMEM/F12) supplemented with 5% FBS and antibiotics. The PEDV strains used in this study were CV777-like strain GDS09 (GI subtype, Genbank ID: MH726408.1) and highly pathogenic variant strain GDS01 (GII subtype, Genbank ID: KM089829.1). Exogenous trypsin (10 μg/mL) was added to proliferate PEDV strains in Vero cells and 5 μg/mL trypsin was added in IPEC-J2 cells. SBTI (soy bean trypsin inhibitor type I; Sigma No. T6522). The endocytic inhibitors used included dynasore (Sigma-Aldrich, No. 324410), chlorpromazine (CPZ, Sigma-Aldrich, No. C0982), methyl-β-cyclodextrin (MβCD, Sigma-Aldrich, No. C4555), nystatin (Sigma-Aldrich, No. 475914), ammonium chloride (NH4Cl, Sigma-Aldrich, No. A9434), and bafilomycin A1 (Baf A1, Sigma-Aldrich, No. 196000). Antibodies against clathrin heavy chain, caveolin 1, EEA1, Rab7, and LAMP1 coupled with secondary goat anti-rabbit Alexa Fluor 488 and goat anti-mouse Alexa Fluor 594 were purchased from Abcam. The mouse anti-PEDV-S monoclonal antibody [34] and anti-PEDV-N polyclonal antibody (prepared in our laboratory) were used in the immunofluorescence analysis and Western blotting analysis, respectively. The overexpression plasmids of wild-type and mutant dynamin II (GFP-Dyn-WT and GFP-Dyn-M), EPS 15 (GFP-EPS15-WT and GFP-EPS15-M), and caveolin-1 (GFP-Cav-WT and GFP-Cav-M) were provided by Prof. Mark McNiven, Mayo Center for Biomedical Discovery (Rochester, MN, USA).

### Dependence of PEDV on trypsin

To investigate the trypsin dependency of PEDV strains, Vero cells were seeded in 6-well plates until confluence. After washed with PBS, cells were infected with PEDV strains at a multiplicity of infection (MOI) of 0.5 with or without trypsin (10 μg/mL) or with trypsin and 25 μg/mL SBTI for 12 h before the quantification of the viruses by qRT-PCR.

### Dynamics of PEDV internalization

To test the dynamics of PEDV internalization, Vero or IPEC-J2 cells were seeded in 12-well plates until confluence. The cells were pre-chilled for 10 min and inoculated with PEDV at a MOI of 0.5 at 4 °C for 1 h for virus binding. The cells were washed three times with ice-cold PBS to remove unbounded viruses and immediately warmed to 37 °C to initiate internalization. After incubation for the indicated time intervals, the cells were treated with proteinase K (1 mg/mL) at 4 °C for 30 min and then washed with PBS to inactivate and remove the non-internalized PEDV particles. The control cells were then washed with PBS. The cells were collected and subjected to qRT-PCR analysis [34].

### Cytotoxicity test and drug treatments

To test the effect of inhibitors on PEDV internalization, it was necessary to evaluate the cytotoxicity of cell inhibitors. The cells were seeded in 96-well plates at a density of 2 × 10^5^ cell/well, grown for 24 h, and treated with endocytic inhibitors at the indicated concentration for 4 h. Then 10 μL of CCK-8 solution was added to each well and incubated at 37 °C for 1 h. An absorbance of 450 nm was measured. The experiments were repeated three times independently. The concentration of each used inhibitor did not cause significant cytotoxicity to the cell viability. To test the effect of inhibitors on PEDV internalization, the cells were pre-treated with different concentrations of drugs for 1 h and then infected with GDS01 or GDS09 strains at MOI = 1 in the presence of drugs for 1 h. After washing with citrate buffer (pH 3.0) [35] and PBS, the cells were incubated with medium containing trypsin for 6 h or 9 h at 37 °C and collected for qRT-PCR and Western blotting analysis, respectively.

### qRT-PCR and Western blotting

The expression of PEDV N protein was detected by qRT-PCR and Western blotting with GAPDH as the reference. Total RNA was extracted using TRIzol (Invitrogen) according to the manufacturer’s instruction and cDNA was synthesized with a ReverTra Ace qPCR RT Master Mix with gDNA Remover Kit (Toyobo, Osaka, Japan). qPCR reaction was performed using a SYBR Premix Ex Taq II Kit (Takara, Tokyo, Japan) using a Light Cycler 480 real-time PCR system (Roche Diagnostics, Indianapolis, IN, USA). For the Western blotting analysis [36], the cells were washed with PBS and lysed in RIPA lysis buffer on ice for 30 min. After SDS-PAGE electrophoresis, proteins were transferred onto polyvinylidene fluoride (PVDF) membrane via the semidry method and immunoblotted with the corresponding antibodies.

### Plasmid and siRNA transfection

Transfection of Vero cells and IPEC-J2 cells with the overexpression plasmids of wild-type and mutant dynamin II (GFP-Dyn-WT and GFP-Dyn-M), EPS 15 (GFP-EPS15-WT and GFP-EPS15-M), and caveolin-1 (GFP-Cav-WT and GFP-Cav-M) were performed using Lipofectamine 2000 (Invitrogen) transfection reagents according to the manufacturer’s protocol. The cells were seeded in 12-well plates until 80% confluence. 24 h after transfection, the cells were infected with PEDV at MOI = 1 for 1 h. Virus was moved with citrate buffer and PBS and replaced with fresh medium containing trypsin, and virus internalization was evaluated by confocal fluorescence microscope.

For the RNA interference assay, siRNAs against dynamin II (siDyn, *Sus scrofa*: 5′-CACCTCATGATCAATAACA-3′, *Chlorocebus sabaeus* 5′-CCTACATCAACACGAACCA-3′), clathrin heavy chain (siCHC, *Sus scrofa* 5′-CCCATACCATGACTGATGA-3′, *Chlorocebus sabaeus* 5′-GATGAACCTTATGCATGCA-3′), EPS 15 (siEPS15, *Sus scrofa* 5′-CCTGTGGATATTCTTGGAA-3′, *Chlorocebus sabaeus* 5′-CCCAGAAACAGCAAGTACA-3′), and caveolin-1 (siCav, *Sus scrofa* 5′-CAACATGCAGAAAGAAATA-3′, *Chlorocebus sabaeus* 5′-CCTTCACTGTGACGAAGTA-3′) were designed and synthesized based on the corresponding full-length mRNA sequences of *Sus scrofa* and *Chlorocebus sabaeus*, siRNAs against Rab7A (siRab7, 5′-GATGGTGGATGACAGACTA-3′), and VPS39 (siVPS39, 5′-GCTTCAAGAGAGACTACTA-3′) were designed and synthesized based on the corresponding mRNA homologous sequences of *Sus scrofa* and *Chlorocebus sabaeus*. The control siRNA (siControl) was designed and synthesized irrelevantly to all the known genes of *Sus scrofa* and *Chlorocebus sabaeus* genome, respectively, by RiboBio (Guangzhou, China). The cells were seeded in 12-well plates until 80% confluence. To ensure transfection efficiency, a second transfection was carried out at 24 h after the first transfection. At 48 h post-first transfection, the cells were infected with PEDV at MOI = 1 for 1 h. Virus was moved with citrate buffer and PBS and replaced with fresh medium containing trypsin, and virus internalization was evaluated by qRT-PCR and Western blotting at 6 hpi and 9 hpi, respectively.

### Co-inoculation of cells with PEDV and transferrin or CTB

Alexa-594 labeled transferrin (Trf) or Alexa-555 labeled cholera toxin B subunit (CTB) were diluted at 1:500 and mixed with PEDV at MOI = 10. The cells were washed three times with PBS and added to the mixture of PEDV and Trf or CTB at 4 °C for 1 h and then incubated at 37 °C for 30 min for internalization. After washing with PBS, the cells were fixed, permeabilized, blocked, incubated with mouse anti-PEDV-S monoclonal antibody, incubated with Alexa 488-conjugated goat anti-mouse IgG (H + L), stained with DAPI, and analyzed using a confocal fluorescence microscope. Light exposure was avoided throughout this experiment.

### Confocal microscopy

Cells cultured in glass-bottom dishes for 12 h were washed with ice-cold PBS and incubated with PEDV at 4 °C for 1 h. Cold viruses were replaced with pre-warmed medium, and the cells were immediately shifted to 37 °C. At specific time points, the cells were fixed in 4% paraformaldehyde at RT for 15 min after washing three times with PBS. Permeabilization was carried with 0.5% Triton X-100 at RT for 15 min. After washing with PBS, the cells were blocked with 5% BSA in PBST at RT for 60 min to block unspecific binding sites. The specific primary antibodies against CHC, EEA1, caveolin-1, Rab7, LAMP1, and anti-PEDV-S antibody were used to probe the cells at 4 °C overnight. The cells were incubated with secondary antibodies (goat anti-rabbit IgG antibody conjugated to Alexa Fluor 488 and goat anti-mouse IgG antibody conjugated to Alexa Fluor 594) at 37 °C for 1 h. Fluorescent images were acquired using the light-scanning module of a Leica TCS SP8 STED 3× confocal microscope.

### Lipid raft isolation

The cells (5 × 10^7^) were incubated or not incubated with PEDV at 37 °C for 1 h, washed three times with ice-cold PBS, and lysed in 1 mL TNE buffer (25 mM Tris, 150 mM NaCl, 5 mM EDTA, and pH 7.5) containing 1% Triton X-100 and 1% phenylmethanesulfonyl fluoride (PMSF) on ice for 30 min. The homogenized cell lysates were centrifuged at 4 °C for 5 min at 1000 *g* and the supernatant was mixed with isometric 1 mL containing 80% sucrose in TNE buffer. The lysates-sucrose mixture was placed at the bottom of ultracentrifugal tubes and overlaid with 7 mL 30% and 3 mL 5% sucrose in TNE buffer. The cell lysates were ultracentrifuged at 4 °C for 16 h at 20 000 *g* in a SW41 rotor (Beckman). After centrifugation, twelve 1 mL fractions were collected from the top to the bottom of the tubes. The fractions were concentrated with 6% PEG at 4 °C overnight, and the pellets were resuspended in 100 μL of TNE buffer after centrifuging at 4 °C for 30 min at 10 000 *g*. The localizations of lipid raft-associated protein caveolin-1 and PEDV N protein were analyzed by Western blotting.

### Image and statistical analyses

All the graphs were created with GraphPad Prism 6 software. All the data are presented as the means ± standard deviations (SDs) from at least three independent experiments. Significance was estimated using one-way ANOVA with multiple comparisons to control. *P* values less than 0.05 were defined as the threshold for statistical significance. *P* values between 0.05 and 0.01 were marked with one asterisk, *P* values between 0.01 and 0.001 were marked with two asterisks, *P* values between 0.001 and 0.0001 were marked with three asterisks, and *P* values less than 0.0001 were marked with four asterisks.

## Results

### Dependence of PEDV on trypsin

**Figure 1:**
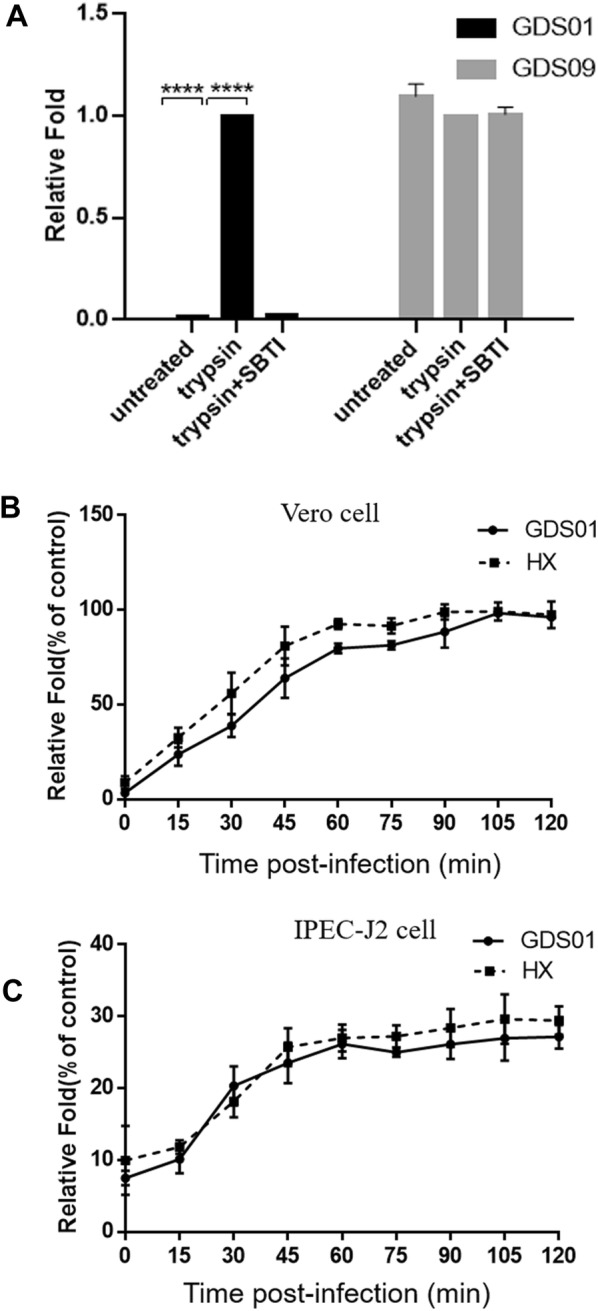
**Trypsin-dependency and kinetics of PEDV entry into cells. A** Vero cells were seeded in 6-well plates until confluence. Cells were washed with PBS and infected with PEDV strains (MOI = 0.5) without trypsin or in the presence of trypsin (10 μg/mL) or trypsin and 25 μg/mL SBTI. Cells were collected for qRT-PCR at 12 hpi. **B**, **C** Vero cells (**B**) and IPEC-J2 cells (**C**) were incubated with PEDV GDS01 and GDS09 strains, respectively, at 4 °C for 1 h and shifted to 37 °C immediately to initiate internalization. At 0, 15, 30, 45, 60, 75, 90, 105, and 120 min after incubation, the cells were treated with proteinase K (1 mg/mL) at 4 °C for 30 min to inactivate the non-internalized virions. The control cells were washed with PBS. The invasion rates were calculated by qRT-PCR analysis. ****P < 0.001.

Coronavirus entry is inextricably linked with proteolytic processing of the S protein. In most cases, PEDV is trypsin dependent. Thus, we investigated the trypsin dependency of both strains used in our research. As shown in Figure 1A, GDS01 strain needed trypsin while GDS09 strain is trypsin independent. So, we added trypsin in the following assays to explore the invasion mechanism of PEDV.

### Kinetics of PEDV internalization

The dynamics of viruses invading different kinds of cells vary, and there may be differences among various subtypes of the same virus. Thus, it is necessary to know the entry dynamics of PEDV before studying the endocytic pathways. The cells were incubated with PEDV at MOI = 0.5 at 4 °C for adsorption and shifted to 37 °C to initiate internalization. The adsorbed but not internalized virions were removed with proteinase K, and the PEDV invasion rates at different time points were detected using qRT-PCR. The invasion kinetics (Figure 1) showed that most of the PEDV particles were detached from the cells by proteinase K at the beginning of invasion. After 60 min in the Vero cells (Figure 1B) and 45 min in the IPEC-J2 cells (Figure 1C), nearly maximum proportions of viral particles completed the internalization. Approximately 95% of the PEDV particles entered the Vero cells, while only 30% entered the IPEC-J2 cells. Notably, the GDS01 strain demonstrated less efficient invasion than the GDS09 strain, but there was no significant difference.

### PEDV entry involves dynamin II

**Figure 2:**
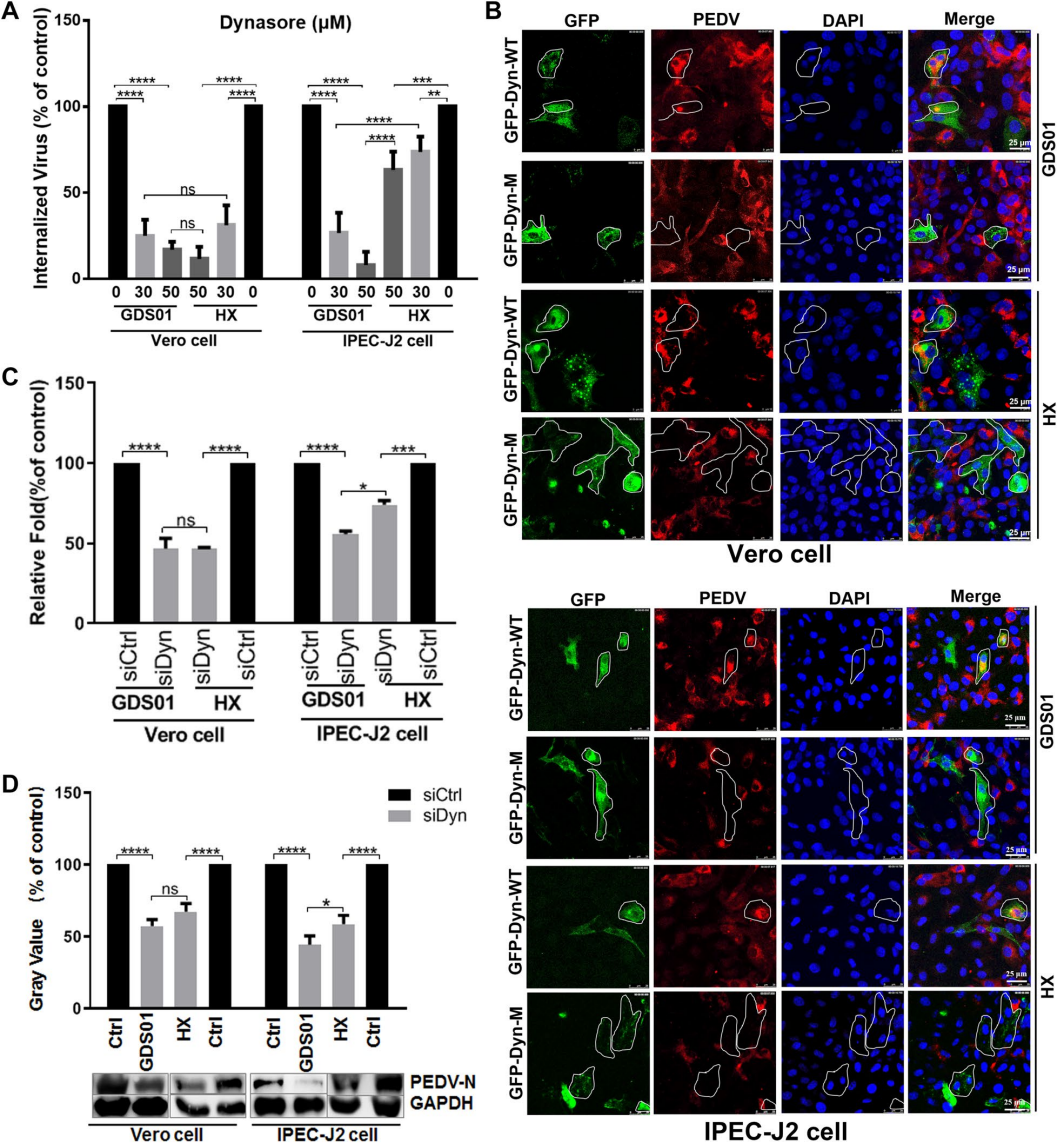
**Dynamin II involved in PEDV entry. A** Cells were pre-treated with 30 μM and 50 μM dynasore at 37 °C for 1 h, respectively, and incubated with GDS01 or GDS09 strain for 1 h. DMSO was used as a negative control. The cells were collected at 6 hpi for qRT-PCR assay to test the invasion efficiency of PEDV. **B** Vero and IPEC-J2 cells were transfected with GFP-Dyn-WT and GFP-Dyn-M, respectively, and infected with PEDV strains at 24 h after transfection. The cells were fixed at 12 hpi and stained for confocal analysis. **C**, **D** Vero and IPEC-J2 cells were transfected with siDyn twice and infected with PEDV strains at 24 h after the second transfection. The invasion rates of PEDV into the cells were detected at 6 hpi and 9 hpi for qRT-PCR and Western blotting analysis, respectively. Ctrl means control. Scale bars indicate 25 μm. **0.05 < *P *< 0.01; ***0.01 < *P *< 0.001; ****P < 0.001.

Dynamin II plays an essential role in cellular membrane fusion during vesicle formation due to its GTPase activity, and it is necessary for clathrin- and caveolae-mediated endocytosis [35, 37]. Thus, we explored the essentiality of dynamin II in PEDV entry using specific chemical inhibitors, overexpression of domain-negative mutants of dynamin II, and siRNA interference. Dynasore [38], a cell-permeable non-competitive inhibitor of dynamin II, was used to pre-treat cells at different concentrations to analyze the effect on PEDV entry. The cytotoxicity test showed that 50 μM of dynasore had no effect on the viability of the Vero and IPEC-J2 cells (Additional file 1). Cells were pre-treated with 30 μM and 50 μM dynasore for 1 h before PEDV infection. DMSO was used as a negative control. The effect of dynasore on PEDV entry was quantified by qRT-PCR at 6 hpi. PEDV invasion was significantly inhibited by dynasore. At a concentration of 50 μM, the invasion rates of the GDS01 and GDS09 strains into the Vero cells were approximately 17% and 12%, and the invasion rates into the IPEC-J2 cells were 8% and 63%, respectively (Figure 2A). A comparison of the invasion rates of the GDS01 and GDS09 strains indicated that the GDS01 strain was more sensitive to dynasore than the GDS09 strain when invading the IPEC-J2 cells but there was no significant difference between them when invading the Vero cells. Many studies used the overexpression of dominant negative mutants to explore the role of dynamin II in virus entry [39, 40]. Mutation of dynamin II from 44K to 44A can inhibit GTPase activity and reduce endocytosis [41]. Cells were transfected with wild-type and mutant types of dynamin II respectively and infected with GDS01 and GDS09 strains at 24 h after transfection. The confocal results showed that the Vero and IPEC-J2 cells overexpressing wild-type dynamin II (GFP-Dyn-WT) were infected with PEDV while the cells overexpressing mutant dynamin II (GFP-Dyn-M) were barely infected (Figure 2B). siRNA interference was also used to identify the importance of dynamin II on virus entry [23, 35, 42]. *Sus scrofa* and *Chlorocebus sabaeus* siRNAs of dynamin II (siDyn) were designed and synthesized. The interference efficiency of siRNA on the dynamin II expression in the Vero and IPEC-J2 cells was obvious at both the mRNA and protein levels (Additional file 1). Cells were infected with PEDV after transfection twice and the internalized virions were quantified at 6 hpi and 9 hpi by qRT-PCR and Western blotting assay, respectively. The qRT-PCR results (Figure 2C) showed that the knockdown of dynamin II expression reduced the PEDV internalization. The internalization rates of the GDS01 and GDS09 strains into Vero cells were approximately 50% and 57%, the internalization rates into the IPEC-J2 cells were approximately 48% and 60%, respectively, but there was no significant difference between the GDS01 and GDS09 strains in the two cells (Figure 2C). The same results were confirmed by Western blotting (Figure 2D). Taken together, the results suggested that PEDV entry relies on dynamin II.

### PEDV entry involves clathrin-mediated endocytosis

**Figure 3:**
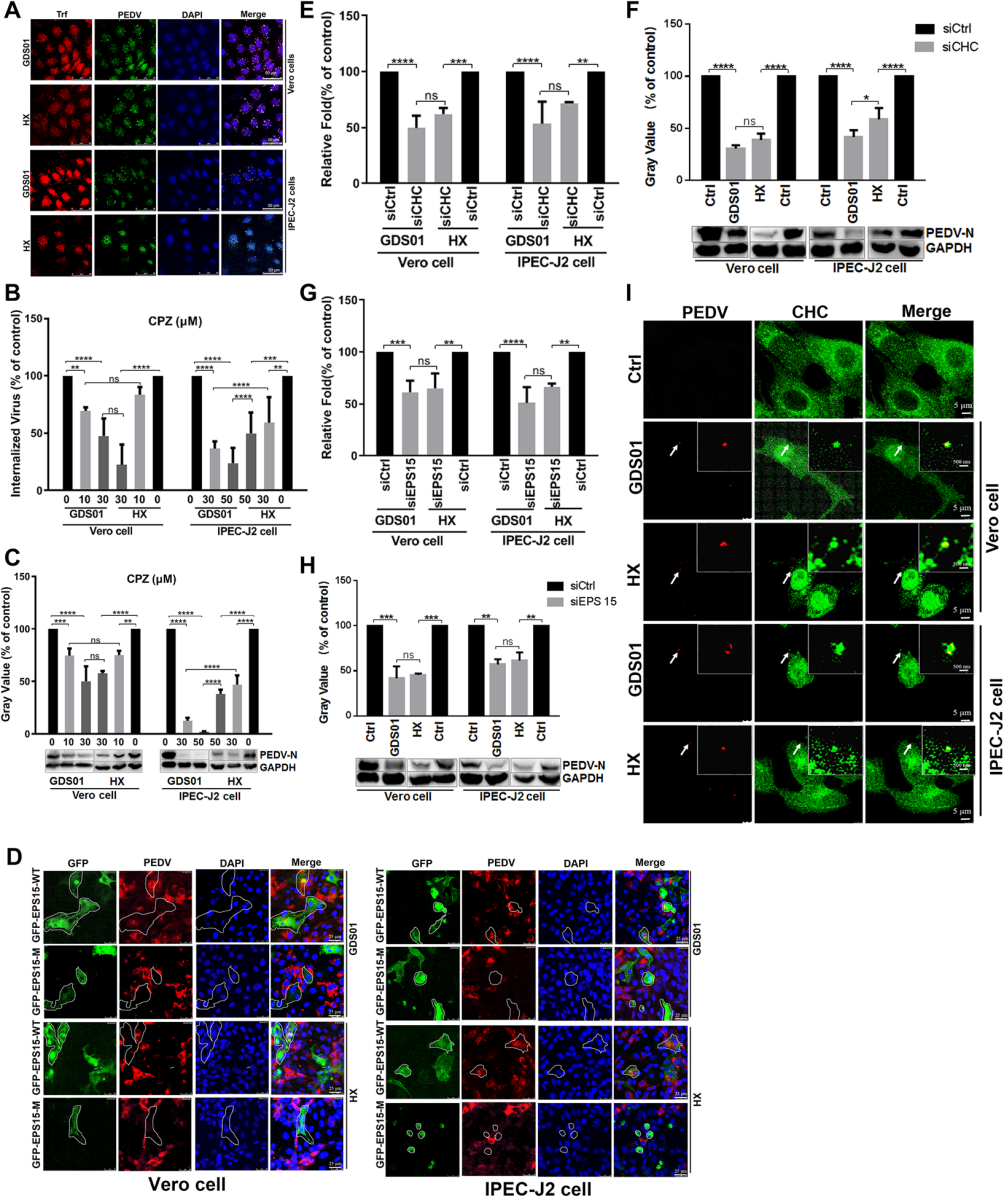
**PEDV entry relies on the CME pathway. A** Vero cells and IPEC-J2 cells were incubated with mixture of Alexa-594 labeled Trf (red) and PEDV (green) at 4 °C for 1 h, and then shifted to 37 °C for 30 min. The cells were fixed and stained for PEDV using monoclonal antibody against PEDV S protein. The cellular localizations of Trf and PEDV were observed with a confocal fluorescence microscope. Light exposure was avoided throughout this process. **B**, **C**. The Vero cells were pre-treated with 10 μM and 30 μM of CPZ, and the IPEC-J2 cells were pre-treated with 30 μM and 50 μM of CPZ, respectively, at 37 °C for 1 h and incubated with GDS01 or GDS09 strains for 1 h. Double-distilled water was used as a negative control. The cells were collected at 6 hpi and 9 hpi for qRT-PCR and Western blotting assay, respectively, to test the invasion efficiency of PEDV. **D** The Vero cells (left) and IPEC-J2 cells (right) were transfected with GFP-EPS15-WT and GFP-EPS15-M, respectively, and infected with PEDV strains at 24 h after transfection. The cells were fixed at 12 hpi and stained for confocal analysis. **E**–**H** The Vero cells and IPEC-J2 cells were transfected with siCHC and siEPS 15 and infected with PEDV strains at 24 h after the second transfection. The cells were collected at 6 hpi and 9 hpi for qRT-PCR and Western blotting analysis, respectively. **I** The cells were pre-cooled at 4 °C for 15 min, incubated with PEDV strains at 4 °C for 1 h, shifted to 37 °C for 5 min to initiate internalization, and washed for three times to remove un-internalized viral particles. The cells were fixed and stained with anti-PEDV-S (red) and anti-CHC (green) primary antibodies. Ctrl means control. Scale bars indicate 50 μm in **A**, 25 μm in **D**, and 5 μm in **I**. **0.05 < *P *< 0.01; ***0.01 < *P *< 0.001; ****P < 0.001.

Clathrin-mediated endocytosis is the most commonly used and classical endocytic pathway for virus entry. To identify whether PEDV utilized CME to enter cells, we co-inoculated the Vero and IPEC-J2 cells with PEDV and Trf, which is the most typical biomolecule that uses CME to enter cells [43]. The co-inoculation results showed that PEDV co-located with Trf in the two types of cells (Figure 3A), which means that PEDV might utilize clathrin-mediated endocytosis to enter cells. To prove this conjecture, we used specific chemical inhibition, the overexpression of domain negative mutants of EPS 15, the knockdown expression of CHC and EPS 15 by siRNA, and the location of PEDV in the cells to estimate the role of clathrin-mediated endocytosis in PEDV entry. CPZ is a specific chemical inhibitor used to block the CME pathway by preventing the assembly of CCPs at the plasma membrane [44]. The cytotoxicity test showed that 30 μM CPZ have no effect on the viability of the Vero cells and 50 μM CPZ have no effect on the viability of the IPEC-J2 cells (Additional file 2). Vero and IPEC-J2 cells were treated with CPZ at different concentrations for 1 h and then infected with PEDV. The internalization rates of PEDV after CPZ treatment were quantified by qRT-PCR and Western blotting at 6 hpi and 9 hpi, respectively. The qRT-PCR results (Figure 3B) showed that PEDV invasion was significantly inhibited by CPZ. The invasion rates of the GDS01 and GDS09 strains at the highest drug concentrations were nearly 48% and 23% in the Vero cells and 24% and 50% in the IPEC-J2 cells, respectively. Notably, there were no significant differences between the GDS01 and GDS09 strains in the Vero cells but the GDS01 strain was more sensitive than the GDS09 strain in the IPEC-J2 cells, reflecting the GDS01 strain’s significantly decreased invasion rates (Figure 3B). The same results were also observed by Western blotting (Figure 3C) and IFA assay (Additional files 3, 4). The role of CME in endocytosis was also identified by the overexpression of GFP-tagged dominant negative mutants of EPS 15 [45]. EPS 15 is a critical component of CCPs by interacting with adaptor protein 2 (AP-2), a major clathrin adaptor complex [46]. Cells transfected with wild-type (GFP-EPS15-WT) and mutant EPS 15 (GFP-EPS15-M) were infected with PEDV strains at 24 h after transfection. The confocal results of the PEDV invasion showed that the cells overexpressing wild-type EPS 15 were infected with PEDV while few infections were observed in the overexpressed GFP-EPS15-M cells (Figure 3D). siRNA was also used to explore the role of CME in PEDV entry by interfering with the expression of clathrin heavy chain (CHC) and EPS 15. CHC and clathrin light chain form a triskelion shape, which is a key component for regulating the formation and disassembly of the clathrin lattice [47]. Cells were infected with PEDV strains after transfection twice, and the invasion rates of the viruses were assessed using qRT-PCR and Western blotting assay at 6 hpi and 9 hpi, respectively. The quantitative experiments showed that knockdown of the expression of CHC significantly reduced the invasion rates of PEDV. The invasion rates of the GDS01 and GDS09 strains were 50% and 62% in the Vero cells and 61% and 65% in the IPEC-J2 cells, respectively, and there was no significant difference between the GDS01 and GDS09 strains (Figure 3E). Knockdown of the expression of EPS 15 also significantly reduced the invasion rates of PEDV. The invasion rates of the GDS01 and GDS09 strains were 61% and 65% in the Vero cells and 51% and 66% in the IPEC-J2 cells, respectively, and there was no significant difference between the GDS01 and GDS09 strains (Figure 3G). The significant inhibition of siCHC and siEPS15 on PEDV entry was also observed by Western blotting assay (Figures 3F, H). To estimate whether PEDV directly entered the cells through CME, we analyzed the localization of PEDV and CHC in the Vero and IPEC-J2 cells, respectively. Pre-cooled cells were incubated with PEDV at 4 °C for 1 h for adsorption and shifted to 37 °C for internalization. Five min later, the cells were washed and fixed for observation using an ultrahigh-resolution laser confocal microscope. The confocal results showed that PEDV particles co-located with CHC protein in the Vero and IPEC-J2 cells (Figure 3I), but some virions were not co-localized with CHC. The results indicated that PEDV can enter cells through the CME pathway, but CME may not be the only pathway utilized by PEDV.

### PEDV entry relies on cholesterol

**Figure 4:**
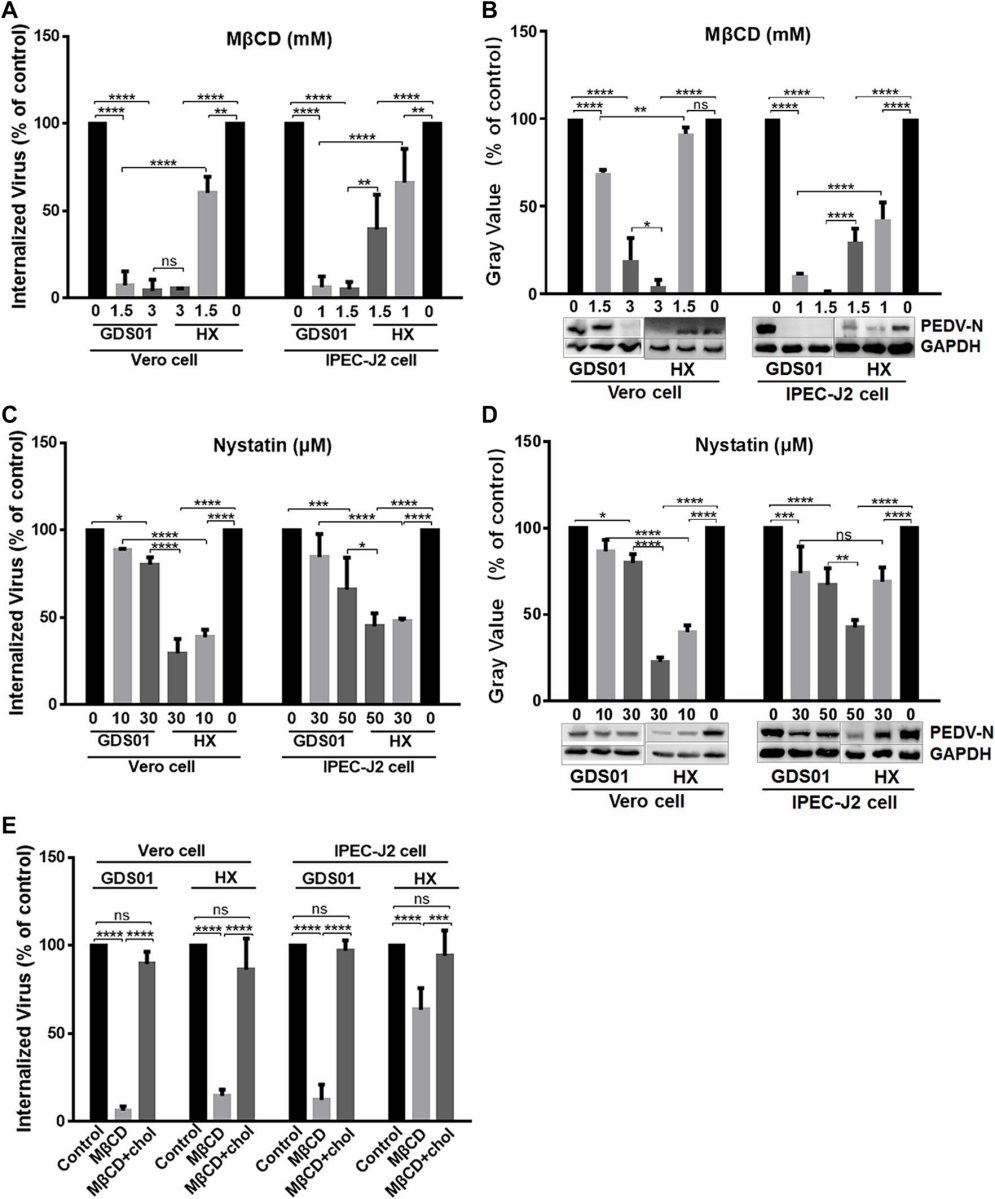
**PEDV entry relies on cholesterol. A**, **B** Vero cells and IPEC-J2 cells were pre-treated with 1.5 mM and 3 mM and 1 mM and 1.5 mM MβCD, respectively, at 37 °C for 1 h and incubated with GDS01 or GDS09 strains for 1 h. Double-distilled water was used as a negative control. The cells were collected at 6 hpi and 9 hpi for qRT-PCR and Western blotting assay, respectively, to test the invasion efficiency of PEDV. **C**, **D** The Vero cells and IPEC-J2 cells were pre-treated with 10 μM and 30 μM and 30 μM and 50 μM of nystatin, respectively, at 37 °C for 1 h and incubated with GDS01 or GDS09 strains for 1 h. DMSO was used as a negative control. The cells were collected at 6 hpi and 9 hpi for qRT-PCR and Western blotting assay, respectively, to test the invasion efficiency of PEDV. **E** The Vero cells and IPEC-J2 cells were pre-treated with different concentrations of MβCD at 37 °C for 1 h, supplemented with 400 μg/mL of soluble cholesterol at 37 °C for 1 h, and infected with PEDV strains for 1 h. The cells were collected at 6 hpi for qRT-PCR assay to test the invasion efficiency of PEDV. *P < 0.05; **0.05 < *P *< 0.01; ***0.01 < *P *< 0.001; ****P < 0.001.

Cholesterol, an important component of cell membranes, embeds phospholipid bilayers and plays a crucial role in the fluidity of cell membranes [48]. Many studies showed that most enveloped virus relied on cholesterol to invade cells [49, 50]. If a virus invades cells, depending on the presence of cholesterol, it will be sensitive to cholesterol extractants. MβCD can eliminate cholesterol on the plasma membrane of cells [51]. Nystatin can bind to the cholesterol-enriched regions of cell membrane and then decompose cholesterol and impair cholesterol synthesis [52]. The cytotoxicity test showed that the maximum tolerance concentrations of Vero and IPEC-J2 cells to MβCD were 3 mM and 1.5 mM, respectively, and the maximum tolerance concentrations to nystatin were 30 μM and 50 μM, respectively (Additional file 5). Cells were pre-treated with different concentrations of MβCD and nystatin for 1 h, then infected with PEDV. The effects of drugs on PEDV entry were estimated by qRT-PCR and Western blotting at 6 hpi and 9 hpi, respectively. MβCD showed a significant inhibition of PEDV entry. The internalization rates of the GDS01 and GDS09 strains after MβCD treatment were approximately 4% and 6% in the Vero cells and approximately 5% and 35% in the IPEC-J2 cells. There were no significant differences between the GDS01 and GDS09 strains in the MβCD-treated Vero cells but in the IPEC-J2 cells, the GDS01 strain was more sensitive to MβCD (Figure 4A). The results were confirmed by Western blotting (Figure 4B) and IFA assay (Additional files 3 and 4). PEDV entry was significantly inhibited by nystatin treatment. The internalization rates of the GDS01 and GDS09 strains after nystatin treatment were approximately 75% and 25% in the Vero cells and approximately 65% and 45% in the IPEC-J2 cells. The GDS09 strain was more sensitive to nystatin than the GDS01 strain in both the Vero and IPEC-J2 cells (Figure 4C). The same results were confirmed by Western blotting (Figure 4D) and IFA assay (Additional files 3 and 4). To further evaluate the importance of cholesterol, cells pre-treated with MβCD were supplemented with exogenous cholesterol and then infected with PEDV, and the changes in the viral invasion rates were quantified by qRT-PCR at 6 hpi. The results showed that adding exogenous cholesterol could significantly increase the invasion rate of PEDV. The average invasion rate of the GDS01 and GDS09 strains increased from 10% to over 90% in the Vero cells and from 10% and 50% to approximately 95% in the IPEC-J2 cells (Figure 4E). The results indicated that PEDV invading and entering cells depended on cholesterol but the two PEDV subtypes showed different degrees of dependence on cholesterol when entering the Vero and IPEC-J2 cells.

### PEDV entry involves caveolae-mediated endocytosis

**Figure 5:**
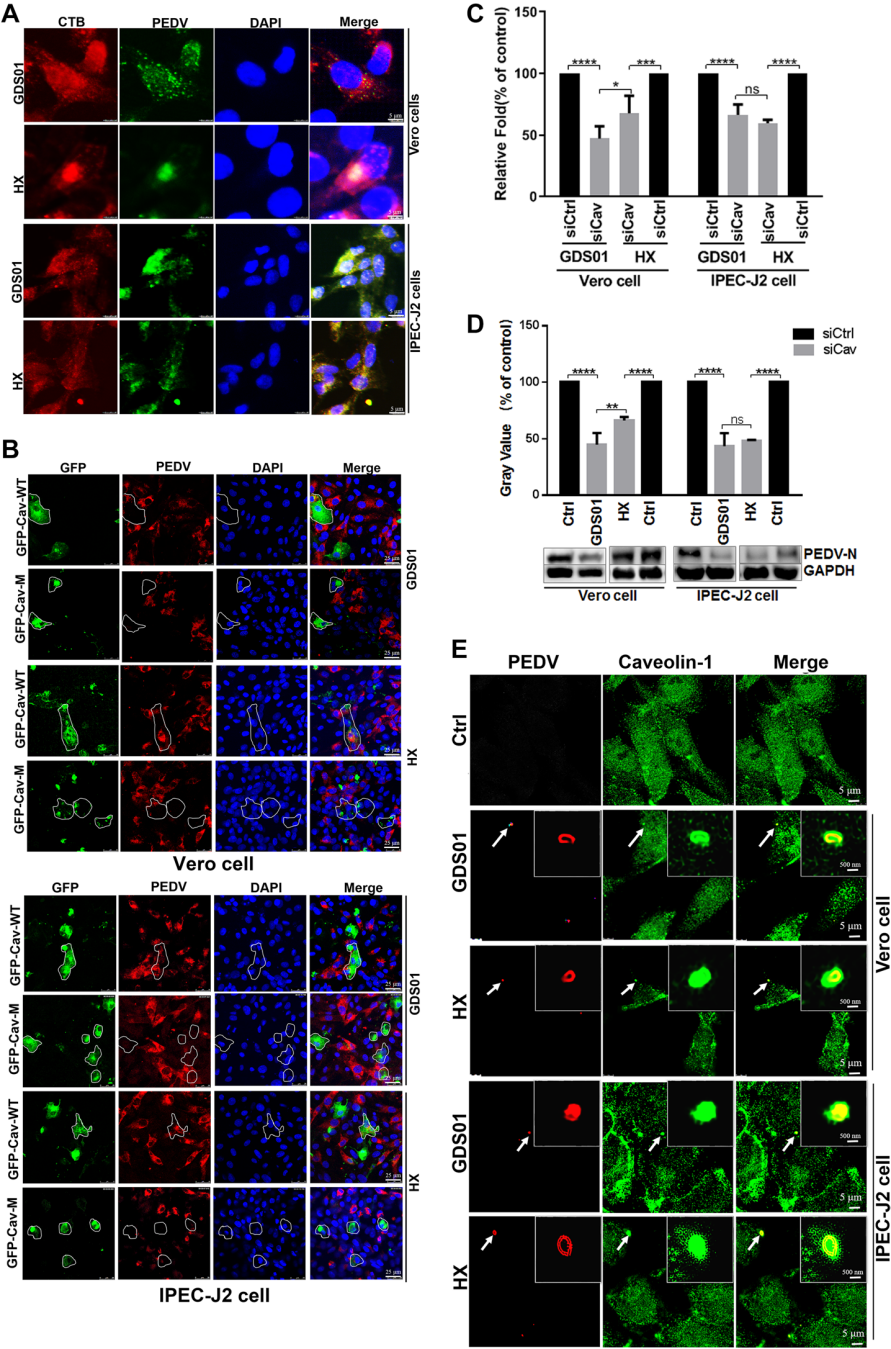
**PEDV enters cells through the caveolae-mediated pathway. A** Vero cells and IPEC-J2 cells were incubated with a mixture of Alexa-555 labeled CTB (red) and PEDV (green) at 4 °C for 1 h, and then shifted to 37 °C for 30 min. The cells were fixed and stained for PEDV using monoclonal antibody against S protein. The cellular localizations of CTB and PEDV were observed with a confocal fluorescence microscope. Light exposure was avoided throughout this process. **B** Vero cells (up) and IPEC-J2 cells (down) were transfected with wild-type caveolin-1 (GFP-Cav-WT) and domain negative mutant of caveolin-1 (GFP-Cav-M), respectively, and infected them with PEDV strains at 24 h after transfection. The cells were fixed at 12 hpi and stained for confocal analysis. **C**, **D** The Vero cells and IPEC-J2 cells were transfected with siCav twice and infected with PEDV strains at 24 h after the second transfection. The cells were collected at 6 hpi and 9 hpi for qRT-PCR and Western blotting analysis, respectively. Ctrl means control. **E** Cells were pre-cooled at 4 °C for 15 min, incubated with PEDV strains at 4 °C for 1 h, shifted to 37 °C to initiate internalization for 10 min, and washed for three times to remove viral particles that were not internalized. The cells were fixed and stained with anti-PEDV-S (red) and anti-caveolin-1 (green) primary antibodies. Scale bars indicate 50 μm in **A**, 25 μm in **B**, and 5 μm in **E**. *P < 0.05; **0.05 < *P *< 0.01; ***0.01 < *P *< 0.001; ****P < 0.001.

As both caveolae and lipid raft are rich in cholesterol, they are sensitive to cholesterol inhibitors [53, 54]. To identify whether caveolae-mediated endocytosis was involved in PEDV entry, cells were co-inoculated with PEDV and CTB, which entered the cells after interactions with specific receptors [55]. The localization results showed that PEDV co-localized with CTB in Vero and IPEC-J2 cells (Figure 5A), which means PEDV may utilize caveolae-mediated endocytosis to enter cells. As the caveolae is mainly coated with caveolin-1 [56], knocking down the expression and separating the interaction factors with caveolin-1 blocked the caveolae-mediated endocytic pathway. Overexpression of the domain-negative mutant of caveolin-1 [57] blocked its interaction with the interaction factors. Cells were transfected with wild-type caveolin-1 (GFP-Cav-WT) and mutant caveolin-1 (GFP-Cav-M), then infected with PEDV at 24 h after transfection, and fixed for confocal observation at 12 hpi. The results showed that PEDV infected GFP-Cav-WT-overexpressing cells but barely infected GFP-Cav-M-overexpressing Vero or IPEC-J2 cells (Figure 5B). siRNAs (siCav) were designed and synthetized to knockdown caveolin-1 expression. Cells were transfected with siCav twice and then infected with PEDV. After the second transfection, the invasion rates of PEDV were measured by qRT-PCR and Western blotting at 6 hpi and 9 hpi, respectively. The qRT-PCR results showed that the knockdown of caveolin-1 expression reduced the internalization of PEDV. The inhibition rates in the GDS01 and GDS09 strains were 53% and 32% in the Vero cells and 33% and 40% in the IPEC-J2 cells, respectively (Figure 5C). Compared with the GDS09 strain, the GDS01 strain showed a higher degree of reduction in the invasion rate in the Vero cells but there was no significant difference in the IPEC-J2 cells (Figure 5C). The same results were confirmed by Western blotting assay (Figure 5D). To identify the role of caveolae in PEDV entry, we investigated the cellular localization of PEDV with caveolin-1. Pre-cooled cells were incubated with PEDV at 4 °C and then shifted to 37 °C for internalization. The cells were then washed and fixed for 10 min for observation with an ultrahigh-resolution laser confocal microscope. The cellular localization results showed that PEDV was co-located with caveolin-1 in the Vero and IPEC-J2 cells (Figure 5E). PEDV can enter cells through the caveolae-mediated pathway.

### PEDV entry involves lipid raft-mediated endocytosis

**Figure 6:**
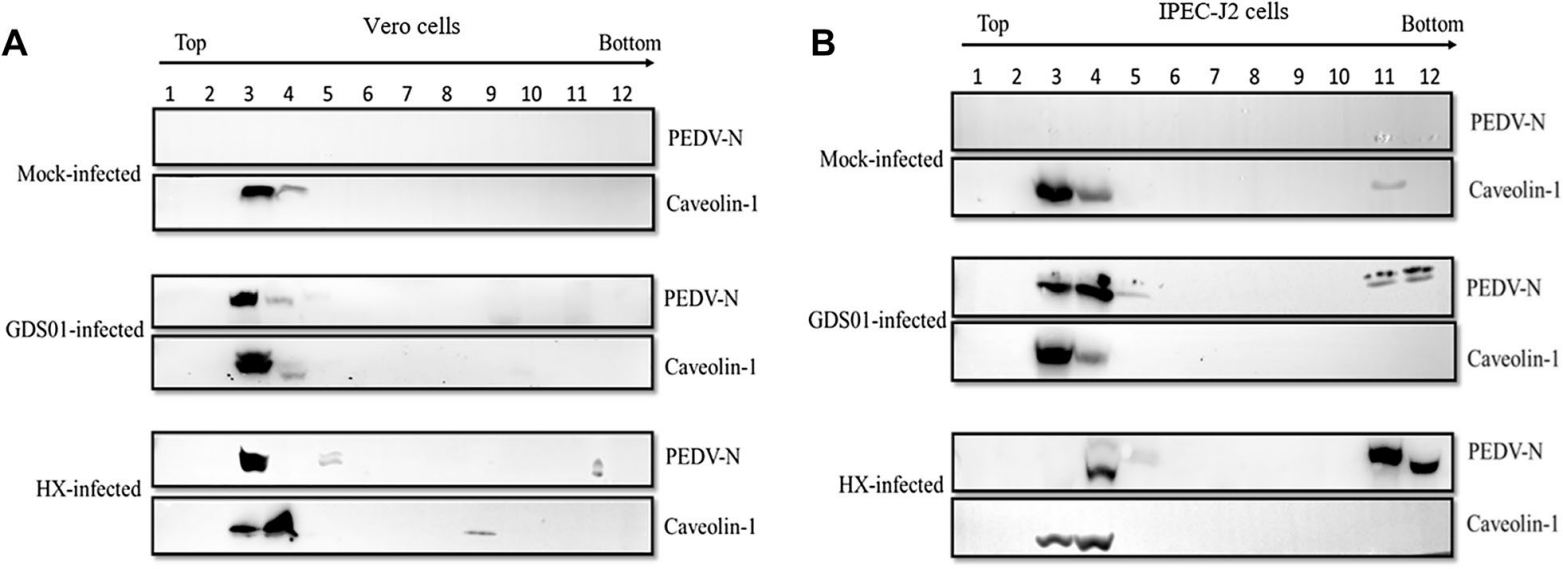
**PEDV utilizes lipid rafts to enter cells.** Vero cells (**A**) and IPEC-J2 cells (**B**) were incubated or not with PEDV at 37 °C for 1 h and then lysed in TNE buffer containing 1% Triton X-100 and 1% PMSF on ice for 30 min. After mixing with isometric 80% sucrose, the homogenized cell lysates were subjected to ultracentrifugation after being overlaid with 30% and 5% sucrose. After centrifugation, a total of 12 fractions were collected from the top to the bottom of the tubes. The localizations of the lipid raft-associated protein caveolin-1 and PEDV N protein were analyzed by Western blotting after being concentrated with 6% PEG6000.

If PEDV can enter cells through the lipid raft pathway, the viral components should be contained in lipid raft enrichment layer after isolated by sucrose density gradient centrifugation [58]. After incubation with PEDV, the cells were lysed and subjected to sucrose gradient centrifugation. The products were extracted from the top down and a total of 12 fractions were obtained for Western blotting analysis. Caveolin-1 was used as the protein marker representing the lipid raft layer [58]. The results showed that PEDV could be detected in the upper lipid raft enrichment layer. Almost all the virions were concentrated in the lipid raft enrichment layer in the Vero cells (Figure 6A) and virions were detected in both the upper and lower components in the IPEC-J2 cells (Figure 6B). The differences between the GDS01 and GDS09 strains were the proportion of virions in the upper and lower components in the IPEC-J2 cells. The GDS01 particles were mainly present in the upper layer while amounts of GDS09 particles were present in the lower layer (Figure 6B). The results indicated that PEDV utilized lipid rafts to enter cells.

### PEDV entry requires low pH

**Figure 7:**
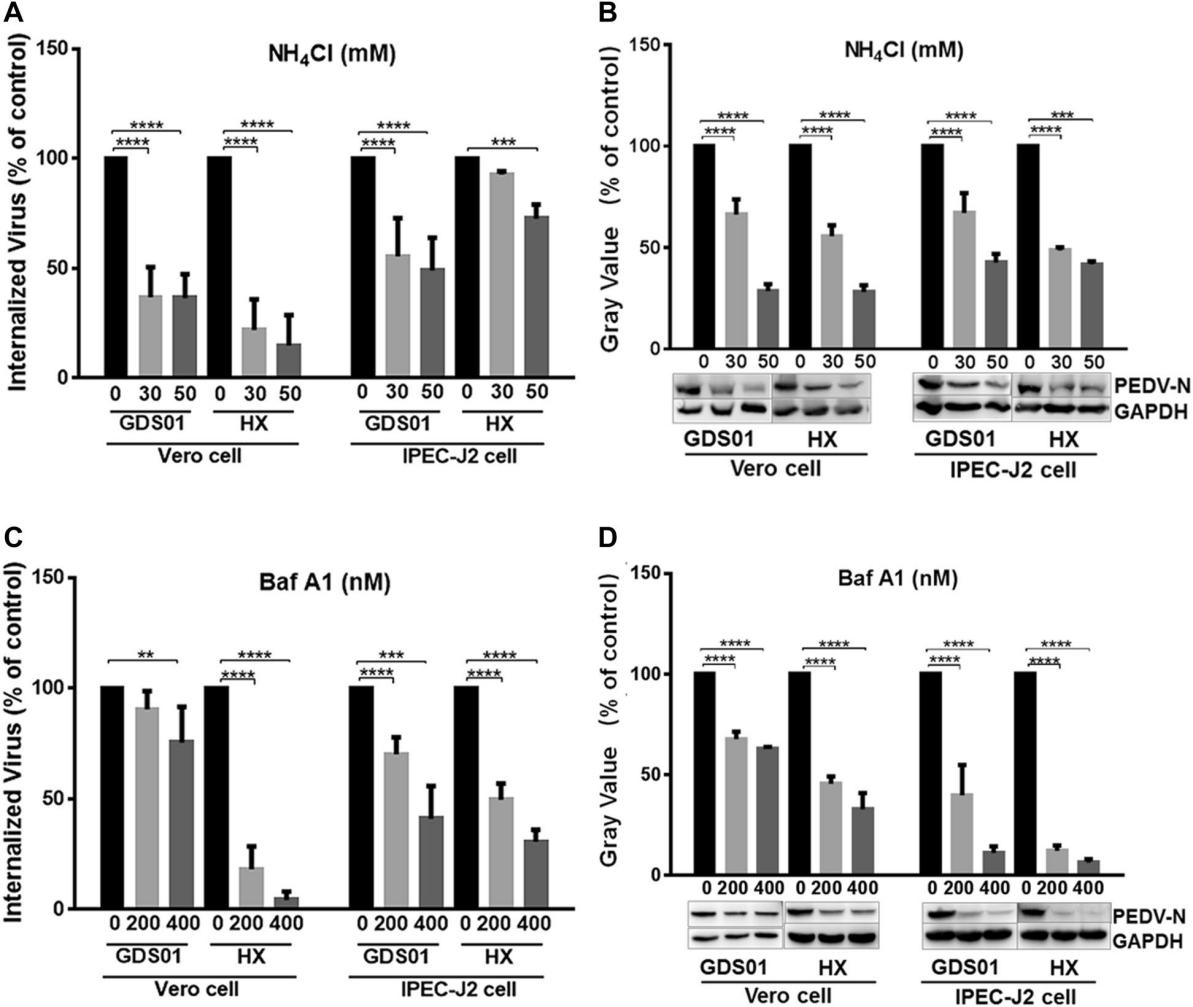
**PEDV entry requires low pH. A**, **B** Vero cells and IPEC-J2 cells were pre-treated with 30 mM and 50 mM NH4Cl at 37 °C for 1 h and then incubated with GDS01 or GDS09 strains for 1 h. Double-distilled water was used as a negative control. The cells were collected 6 hpi and 9 hpi for qRT-PCR and Western blotting assay, respectively, to test the invasion efficiency of PEDV. **C**, **D** The Vero cells and IPEC-J2 cells were pre-treated with 200 nM and 400 nM Baf A1 at 37 °C for 1 h and then incubated with GDS01 or GDS09 strains for 1 h. DMSO was used as a negative control. The cells were collected 6 hpi and 9 hpi for qRT-PCR and Western blotting assay, respectively, to test the invasion efficiency of PEDV. **0.05 < *P *< 0.01; ***0.01 < *P *< 0.001; ****P < 0.001.

Viruses that enter cells via endocytosis are usually trafficked by endocytic vesicles to early endosomes for sorting and are transported to late endosomes or fused with early endosomes [59]. If viruses do not fuse in the early endosomes and release genomes into the cytoplasm, they will enter the late endosomes with the further acidification and maturation of the early endosomes. Similarly, if the pH environment in the late endosomes does not meet the requirements for conformational changes of viral glycoproteins to cause membrane fusion, viruses will enter the lysosomes with the transportation of the late endosomes and finally achieve membrane fusion. Viruses require acidic pH to traffic between endosomes. Therefore, it is necessary to clarify whether PEDV relies on a low pH environment. NH4Cl is an inhibitor of endosome acidification [60]. Baf A1 is a V-ATPase inhibitor that can block the traffic of endocytic cargoes from early endosomes to late endosomes and inhibit the stability of low pH environments in the lysosome lumen [61]. Cells were pre-treated with different concentrations of NH4Cl and Baf A1 (Additional file 6) and infected with PEDV (Additional file 4). The invasion rates of PEDV were measured by qRT-PCR and Western blotting at 6 hpi and 9 hpi, respectively. The results showed that cells pre-treated with NH4Cl significantly inhibited PEDV entry. The invasion rates in the GDS01 and GDS09 strains were approximately 30% and 15% in the Vero cells and 50% and 73% in the IPEC-J2 cells (Figure 7A). This significant inhibition was also confirmed by Western blotting (Figure 7B) and IFA assay (Additional files 3 and 4). Baf A1 can significantly inhibit PEDV entry. The invasion rates in the GDS01 and GDS09 strains were approximately 75% and 5% in the Vero cells and 41% and 30% in the IPEC-J2 cells (Figure 7C). Western blotting (Figure 7D) and IFA assay (Additional files 3, 4) also confirmed the inhibition of Baf A1. The results proved that PEDV entry requires low pH.

### Internalized PEDV is trafficked to lysosomes via endosomes

**Figure 8:**
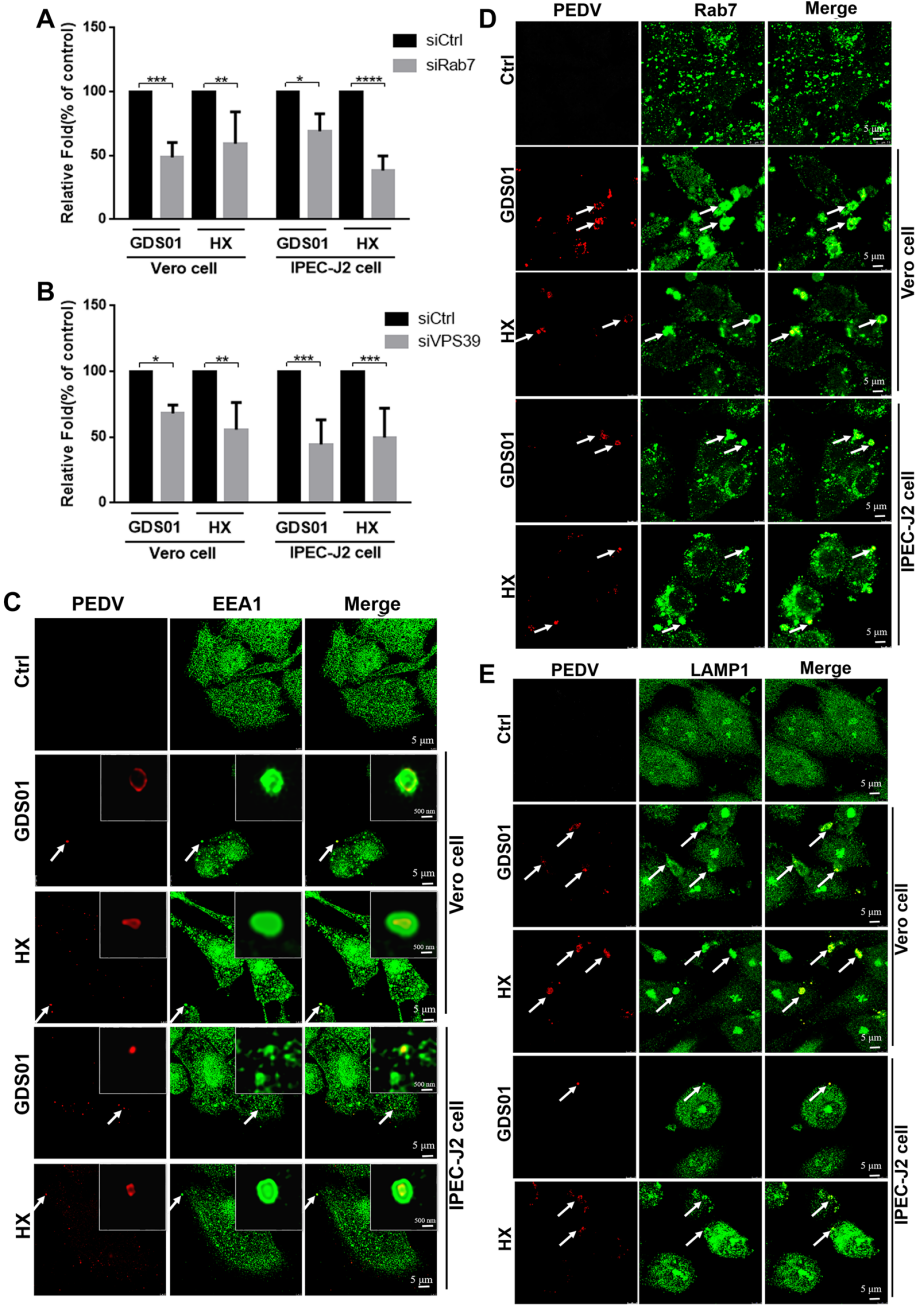
**PEDV traffics to lysosome via endosomes. A**, **B** Vero cells and IPEC-J2 cells were transfected with siRab7 and siVPS39 twice, respectively, and then infected with PEDV strains at 24 h after the second transfection. The cells were collected at 6 hpi for qRT-PCR analysis. Ctrl means control. **C**–**E** The Vero cells and IPEC-J2 cells were pre-cooled at 4 °C for 15 min, incubated with PEDV strains at 4 °C for 1 h, shifted to 37 °C to initiate internalization. The non-internalized viral particles were removed by washing. 30 min after shifting, the cells were fixed and stained with anti-PEDV-S (red) and anti-EEA1 (green) primary antibodies (**C**). 40 min after shifting, the cells were fixed and stained with anti-PEDV-S (red) and anti-Rab7 (green) primary antibodies (**D**). 50 min after shifting, the cells were fixed and stained with anti-PEDV-S (red) and anti-LAMP1 (green) primary antibodies (**E**). Scale bars indicate 5 μm in **C**–**E**. *P < 0.05; **0.05 < *P *< 0.01; ***0.01 < *P *< 0.001; ****P < 0.001.

Early endosomes mature into late endosomes by increasing intraluminal acidity through proton pump activity. Late endosomes can become larger vesicles by fusing with the same type of endosomes, so they mostly exist in the form of a multivesicular body (MVB). Late endosomes release Rab5, incorporate Rab7, and prepare to fuse with lysosomes [62]. To explore whether PEDV particles are trafficked after internalization, we interfered with the expression of Rab7 [63] involved in late endosomes and VPS39 [32] involved in late endosome-to-lysosome maturation and identified whether viral particles co-located with early endosomes, late endosomes and lysosomes. Knockdown of the expression of Rab7 (siRab7) can significantly inhibit the invasion efficiency of PEDV. The invasion rates of the GDS01 and GDS09 strains were 49% and 51% in the Vero cells and 82% and 38% in the IPEC-J2 cells (Figure 8A). Similarly, the knockdown of the expression of VPS39 (siVPS39) also significantly inhibited the invasion efficiency of PEDV. The invasion rates of the GDS01 and GDS09 strains were 68% and 56% in the Vero cells and 45% and 50% in the IPEC-J2 cells (Figure 8B). For cellular location by PEDV observation, pre-cooled cells were incubated with PEDV at 4 °C and then shifted to 37 °C for internalization. The cells were washed and fixed at different time points after shift for observation using an ultrahigh-resolution laser confocal microscope. Cellular localization assays showed that internalized PEDV could co-locate with EEA1, the early endosome protein marker, in the Vero and IPEC-J2 cells 30 min after endocytosis (Figure 8C) and could co-locate with Rab7, the late endosome protein marker, 40 min after endocytosis (Figure 8D), while the late endosomes were mostly in the form of MVB. Co-localization of PEDV with LAMP1 (lysosomal associated membrane protein 1), an important lysosome membrane component, was also observed 50 min after endocytosis in the two types of cells (Figure 8E). The results demonstrated that PEDV was trafficked to the lysosomes after entering the cells through endocytosis, and there were no differences between the two PEDV genotypes and cells.

## Discussion

Since highly pathogenic variant strains emerged in 2010, PEDV has attracted global attention. Many studies have reported that vaccines based on CV777 or CV777-like strains have low protection efficiency against re-emerging variant strains [3, 4, 6–8]. Genotyping showed that PEDV strains can be sorted into two genotypes, GI subtypes (classical) and GII subtypes (variant). The nucleotide sequence of S1 subunit of S protein is 75–90% similar between GI and GII, which shows high variability [10, 64]. As the main antigen of PEDV, the S protein plays an important role in inducing immune responses and viral entry. Mutation of the S gene may lead to different mechanisms of virus invasion, which may help elucidate the pathogenesis and immune evasion of PEDV. As SARS-CoV utilizes varying endocytic routes to invade different cells [23, 24], we wondered whether PEDVs can enter cells through different pathways. Burkard et al. clarified that coronavirus entered cells through the endosome/lysosome pathway and was proteolytic dependent. The furin cleavage site just upstream of the fusion peptide (FP) of the S protein was the key to determining the fusion site of the viral membrane [32]. While the S protein of PEDV does not have the furin cleavage site, a conserved arginine just upstream of the putative FP as the potential cleavage site can be cleaved by trypsin [65]. Whether the theory mentioned above is applicable to PEDV thus needs further study. Here, we explored the GI and GII subtype pathways of PEDV entry into Vero and IPEC-J2 cells, respectively, and the transportation route after internalization. Our results showed that two the subtypes of PEDV utilized clathrin-, caveolae-, and lipid raft-mediated endocytosis to enter the Vero and IPEC-J2 cells, but the utilization efficiency of each endocytic pathway varied depending on the different genotypes and types of cells.

To describe the dynamic curve of PEDV entry, the appropriate viral scavenger is extremely important to remove virus particles effectively adsorbed on the cell surface. We compared the effects of citrate buffer (pH 3.0) with proteinase K (1 mg/mL), and the latter exhibited a stronger capacity to remove viruses. The results of dynamic invasion showed that the virus invaded Vero cells 100% within 60 min, while the invasion efficiency of the IPEC-J2 cells was only approximately 30%. Although IPEC-J2 cells are considered the host cells of PEDV, the cell lines cultured in vitro lost their polar growth state in vivo [66], which possibly affects the viral recognition and reduces the infection efficiency of the virus. GDS01 showed a lower invasion rate than GDS09, but there was no significant difference.

Dynamin II, a GTPase, plays an important role in endocytosis by pinching the endocytic vesicles off the plasma and is necessary in clathrin- and caveolae-mediated endocytosis. We found that PEDV entry is dynamin II-dependent, although the sensitivity of the GDS01 and GDS09 strains to dynasore varied. Considering the low specificity of chemistry inhibitor, dominant negative mutant and siRNA-mediated knockdown of dynamin II were carried out to evaluate dynamin II for PEDV infection. Both the GDS01 and GDS09 strains were inhibited by siDynamin II with no significant difference.

Clathrin-mediated endocytosis is a classical and commonly used pathway for most enveloped viruses. Many coronaviruses use CME to enter cells, such as SARS-CoV entry into HepG2 cells and COS7 cells and PHEV entry into Neuro-2a cells [23, 35]. To ascertain whether PEDV utilized CME to enter cells, the chemistry inhibitor CPZ was used to prevent clathrin assembly and further block CME. Clathrin is composed of light chain (CLC) and heavy chain (CHC) which form clathrin lattices under the interaction of AP-2 and EPS 15. Both CPZ pre-treatment and siRNA-mediated knockdown of CHC and EPS 15 can significantly reduce the invasion rate of PEDV into cells, with no significant difference between the GDS01 and GDS09 strains. Dominant negative mutants can provide a more specific method to study endocytic pathways by separating the prototype protein from their interaction regulatory factors. In this study, we also showed that cells overexpressing dominant negative mutant of EPS 15 were barely infected with PEDV but cells overexpressing wild-type EPS 15 were infected as normal. However, the inhibition of endocytosis by overexpressing dominant negative mutants may be compensated through other clathrin-independent endocytic pathways. When viruses enter cells through CME, they are carried by clathrin-coated vesicles. Co-localization of viral particles and CHC indicated that PEDV entry relies on CME.

Although caveolae and lipid rafts have the same components, such as caveolin-1, GM1, and cholesterol, they are two completely different endocytic pathways. Before investigating whether PEDV can use these two pathways to enter cells, we first examined whether PEDV invasion depends on cholesterol. The cholesterol inhibitors nystatin and MβCD had significant inhibitory effects on PEDV entry. Nystatin had higher inhibitory effects on GDS09 entry than GDS01. The inhibitory effects of MβCD on Vero cells were similar for GDS09 and GDS01 strains. MβCD effects were lower on GDS09 than GDS01 in IPEC-J2 cells. We hypothesized that GDS01 cell invasion mainly depended on cholesterol on the cell surface, while GDS09 depended on the presence of cholesterol on the cell surface and cholesterol synthesis. Exogenous cholesterol supplementation also confirmed the importance of cholesterol in PEDV entry. Endocytic vesicles formed in the caveolae-mediated pathway were coated with caveolin-1, which plays a critical role in the process. Dominant negative mutant, RNA interference, and the cellular co-localization of caveolin-1 with viral particles provided further evidence that PEDV entry needed caveolin-1. Collectively, both subtypes of PEDV entered the Vero and IPEC-J2 cells through caveolae-mediated endocytosis. However, Park et al. [33] showed that PEDV entry was independent of caveolae-coated pit assembly by treating Vero cells with nystatin. The different results may be explained by different operational details. Firstly, nystatin was used before and during the incubation of PEDV in this study, while only before incubation in the research of Park et al. The concentrations of nystatin used in the two studies were different. The highest concentration of nystatin used in Park’s research was 20 μM [33], while the highest concentration we used was 30 μM in Vero cells. When the concentration is 10 μM, nystatin does not inhibit the entry of PEDV, which is consistent with Park’s results. Secondly, Park et al. added methyl cellulose to block second-cycle infection [33], while we added nothing except trypsin in medium. Whether these reasons cause two different results needs further study.

Lipid raft acted as a platform for cell signal transduction and viral invasion, distributed in an island form on the plasma membrane of cells, and was isolated by sucrose density gradient centrifugation. Western blotting analysis showed that PEDV N protein located in the lipid raft (upper layer) with caveolin-1 in the cells. As shown in Figure 6B, PEDV N protein is also present in the bottom layer when infected with IPEC-J2 cells, which may be due to the different composition of the plasma membranes of the two kinds of cells.

We demonstrated that PEDV GI subtype GDS09 and GII subtype GDS01 strains could enter Vero and IPEC-J2 cells via the clathrin-, caveolae-, and lipid raft-mediated endocytosis pathways. Furthermore, we also found that the invasion efficiency of the two strains was different with different endocytosis pathway. These differences between GDS01 and GDS09 strains may be due to the difference of S gene especially the S1 region of S gene (homology was about 92%), which is responsible for cell entry and membrane fusion by binding with receptor. The difference of gene may lead to the difference of binding ability or affinity between S protein and receptor, thus leading to the different utilization or initiation efficiency of different endocytosis pathways. However, whether the different gene sequence causes different invasion efficiency between GDS01 and GDS09 strains needs further study.

After internalization, viral particles are transported by specific endosomes for membrane fusion. The classical transit route is the endo-/lysosomal pathway, in which endocytic cargoes are transported along endocytic vesicles, early endosomes, and late endosomes-lysosomes. Park et al. have confirmed that NH_4_Cl and Baf-A1 could inhibit PEDV entry [33], which is consistent with our results, but needs to be confirmed by different methods. In this study, in addition to chemical inhibitors, we also used siRNA interference and cellular localization of virus particles to identify the role of pH and endosomes. The results of this study revealed that PEDV entry relied on low pH, which means that internalized PEDV particles are transported to endosomes and lysosomes, as demonstrated by the co-localization of viral particles with EEA1, Rab7, and LAMP1. Liu et al. [67] reported that PEDV S protein was activated by lysosomal cysteine proteases to activate PEDV entry. However, based on the data, we could not conclude that membrane fusion occurred at the lysosomes; more technical methods are necessary to demonstrate the mechanism.

In conclusion, studying the internalization and intracellular trafficking mechanism of PEDV are important to understand viral pathogenesis and benefit to the development of future therapies strategies. This study demonstrated that both the GI and GII subtypes of PEDV enter Vero and IPEC-J2 cells via the clathrin-, caveolae-, and lipid raft-mediated endocytosis pathways, but the efficiency of each endocytosis pathway varies depending on the different genotypes and types of cells. The internalized PEDV entered the lysosomes through the early and late endosomes. The results of this study provide a theoretical basis for the further understanding of PEDV pathogenesis to find new targets of antiviral drugs.

## Supplementary information

**Additional file 1. Dynamin II is involved in PEDV entry.** (A) Vero cells and IPEC-J2 cells were treated with different concentrations of dynasore at 37 °C for 4 h. CCK-8 solution was added to each well at 37 °C for 1 h, and absorptions of 450 nm were detected. DMSO was used as a negative control. (B) The Vero cells and IPEC-J2 cells were transfected with siDyn, and the second transfection was carried out at 24 h after the first transfection. The inference efficiency was detected by qRT-PCR and Western blotting at 48 h after the first transfection. Ctrl means control. **0.05 < *P *< 0.01; ***0.01 < *P *< 0.001; ****P < 0.001.

**Additional file 2. Clathrin-mediated endocytosis is involved in PEDV entry.** (A) Vero cells and IPEC-J2 cells were treated with different concentrations of CPZ at 37 °C for 4 h. CCK-8 solution was added to each well at 37 °C for 1 h, and absorptions of 450 nm were detected. Double-distilled water was used as a negative control. (B, C) The Vero cells and IPEC-J2 cells were transfected with siCHC and siEPS15, and the second transfection was carried out at 24 h after the first transfection. The inference efficiency was detected by qRT-PCR and Western blotting at 48 h after the first transfection. Ctrl means control. *P < 0.05; **0.05 < *P *< 0.01; ***0.01 < *P *< 0.001; ****P < 0.001.

**Additional file 3. Endocytic drugs inhibited PEDV entry into Vero cells.** Cells were seeded in 12-well plates until confluence. Cells were pre-treated with 30 μM CPZ, 3 mM MβCD, 30 μM Nystatin, 50 mM NH4Cl and 200 nM Baf A1 respectively, at 37 °C for 1 h and incubated with GDS01 (A) and GDS09 (B) strains for 1 h. Double-distilled water was used as a negative control. The cells were collected at 9 hpi and detected by immunofluorescence staining against the PEDV S protein (green). Nuclei were stained with DAPI (blue). Scale bars indicate 100 μm.

**Additional file 4. Endocytic drugs inhibited PEDV entry into IPEC-J2 cells.** Cells were seeded in 12-well plates until confluence. Cells were pre-treated with 50 μM CPZ, 1.5 mM MβCD, 50 μM Nystatin, 50 mM NH4Cl and 400 nM Baf A1 respectively, at 37 °C for 1 h and incubated with GDS01 (A) and GDS09 (B) strains for 1 h. Double-distilled water was used as a negative control. The cells were collected at 15 hpi and detected by immunofluorescence staining against the PEDV S protein (green). Nuclei were stained with DAPI (blue). Scale bars indicate 100 μm.

**Additional file 5. PEDV entry relies on cholesterol and caveolin-1.** (A, B) Vero cells (A) and IPEC-J2 cells (B) were treated with different concentrations of MβCD at 37 °C for 4 h, respectively. CCK-8 solution was added to each well at 37 °C for 1 h, and absorptions of 450 nm were detected. Double-distilled water was used as a negative control. (C) The Vero cells and IPEC-J2 cells were treated with different concentrations of nystatin at 37 °C for 4 h, respectively. CCK-8 solution was added to each well at 37 °C for 1 h, and absorptions of 450 nm were detected. DMSO was used as a negative control. (D) The Vero cells and IPEC-J2 cells were transfected with siCav, and the second transfection was carried out at 24 h after the first transfection. The inference efficiency was detected by qRT-PCR and Western blotting at 24 h after the second transfection. Ctrl means control. (E) The Vero cells (up) and IPEC-J2 cells (down) were treated with MβCD at 37 °C for 1 h, and the amount of cholesterol was detected following the instructions of the cholesterol quantitative kit (AmyJet Scientific). A histogram was created via the fluorescence density. The error bars represent the SD of 10 figures from three independent experiments. *P < 0.05; **0.05 < *P *< 0.01; ***0.01 < *P *< 0.001; ****P < 0.001.

**Additional file 6. Internalized PEDV traffics to lysosomes via endosomes.** (A, B) Vero cells (A) and IPEC-J2 cells (B) were treated with different concentrations of NH4Cl and Baf A1 at 37 °C for 4 h, respectively. CCK-8 solution was added to each well at 37 °C for 1 h, and absorptions of 450 nm were detected. Double-distilled water and DMSO were used as negative controls, respectively. (C-D) The Vero cells (C) and IPEC-J2 cells (D) were transfected with siRab7 and siVPS39, respectively, and the second transfection was carried out at 24 h after the first transfection. The inference efficiency was detected by qRT-PCR at 24 h after the second transfection. **0.05 < *P *< 0.01; ***0.01 < *P *< 0.001; ****P < 0.001.

## Abbreviations

PEDV: porcine epidemic diarrhea virus
CME: clathrin-mediated endocytosis
CCPs: clathrin-coated pits
CCVs: clathrin-coated vesicles
SARS-CoV: severe acute respiratory syndrome coronavirus
MHV: murine hepatitis virus
HCoVs: human coronavirus
MERS-CoV: middle east respiratory syndrome coronavirus
FIPV: feline infectious peritonitis virus
CHC: clathrin heavy chain
CLC: clathrin light chain
MVB: multivesicular body
LAMP1: lysosomal associated membrane protein 1
CPZ: chlorpromazine
MβCD: methyl-β-cyclodextrin
Baf A1: bafilomycin A1
CTB: cholera toxin B subunit
Trf: transferrin
MOI: multiplicity of infection
PMSF: phenylmethanesulfonyl fluoride
