# Supplementary figures and images for: PEDV enters cells through clathrin-, caveolae-, and lipid raft-mediated endocytosis and traffics via the endo-/lysosome pathway
Source: Vet Res. 2020 Feb 10;51:10. doi: 10.1186/s13567-020-0739-7 (PMC7011528; doi:10.1186/s13567-020-0739-7)

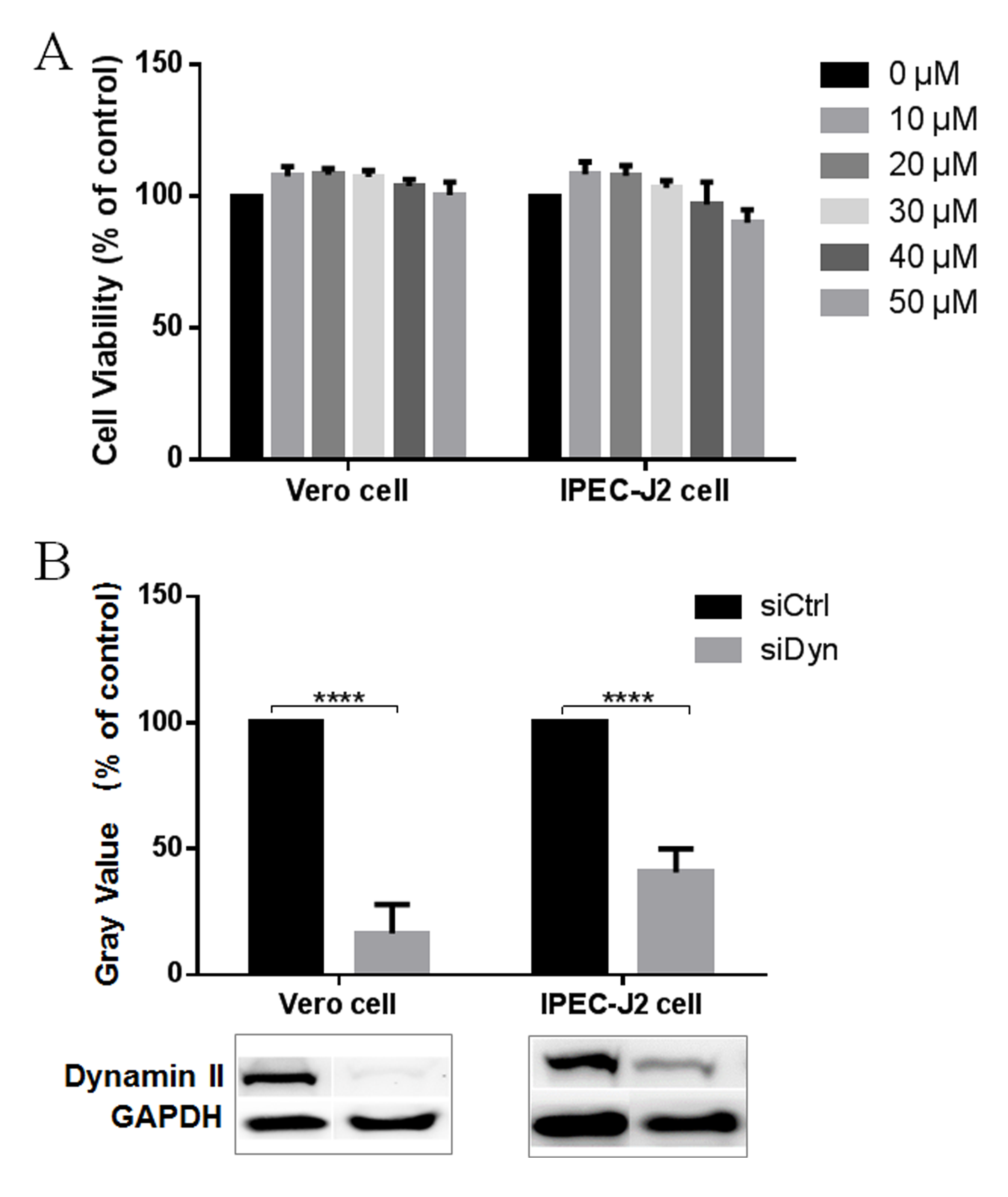

Supplement: Supplementary file 1 — Additional file 1. Dynamin II is involved in PEDV entry. (A) Vero cells and IPEC-J2 cells were treated with different concentrations of dynasore at 37 °C for 4 h. CCK-8 solution was added to each well at 37 °C for 1 h, and absorptions of 450 nm were detected. DMSO was used as a negative control. (B) The Vero cells and IPEC-J2 cells were transfected with siDyn, and the second transfection was carried out at 24 h after the first transfection. The inference efficiency was detected by qRT-PCR and Western blotting at 48 h after the first transfection. Ctrl means control. **0.05 < P < 0.01; ***0.01 < P < 0.001; ****P < 0.001. [file 13567_2020_739_MOESM1_ESM.tif]

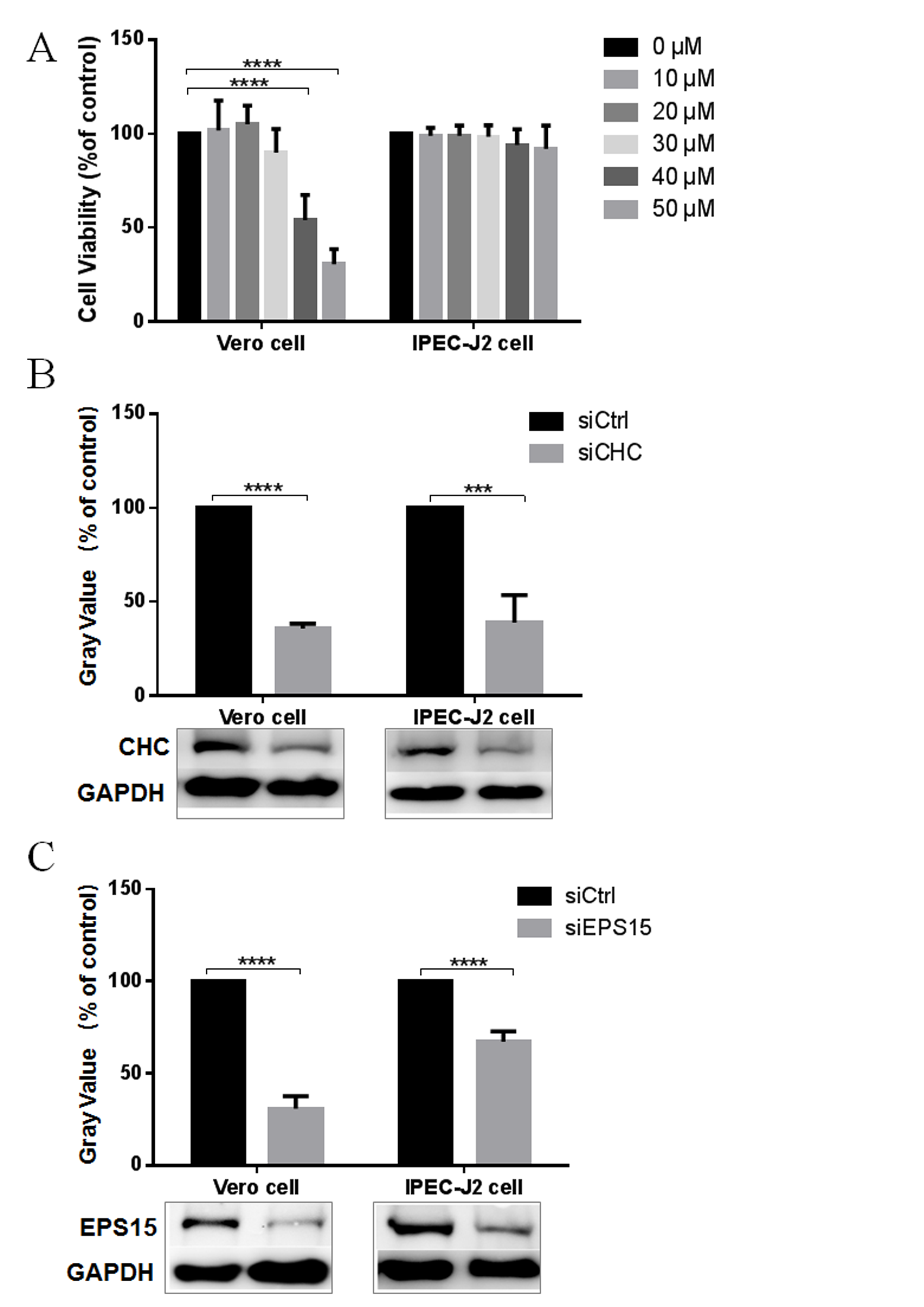

Supplement: Supplementary file 2 — Additional file 2. Clathrin-mediated endocytosis is involved in PEDV entry. (A) Vero cells and IPEC-J2 cells were treated with different concentrations of CPZ at 37 °C for 4 h. CCK-8 solution was added to each well at 37 °C for 1 h, and absorptions of 450 nm were detected. Double-distilled water was used as a negative control. (B, C) The Vero cells and IPEC-J2 cells were transfected with siCHC and siEPS15, and the second transfection was carried out at 24 h after the first transfection. The inference efficiency was detected by qRT-PCR and Western blotting at 48 h after the first transfection. Ctrl means control. *P < 0.05; **0.05 < P < 0.01; ***0.01 < P < 0.001; ****P < 0.001. [file 13567_2020_739_MOESM2_ESM.tif]

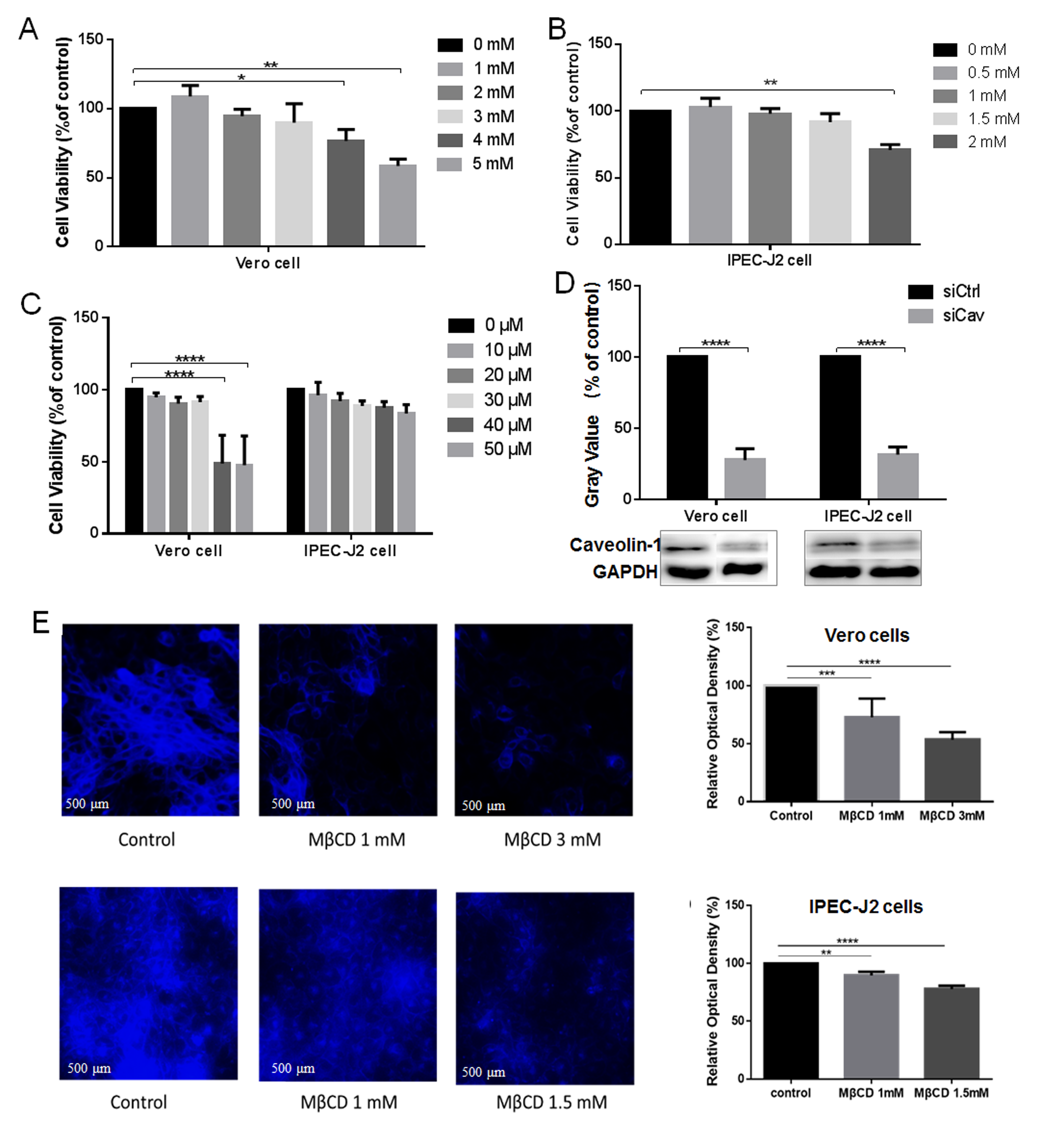

Supplement: Supplementary file 5 — Additional file 5. PEDV entry relies on cholesterol and caveolin-1. (A, B) Vero cells (A) and IPEC-J2 cells (B) were treated with different concentrations of MβCD at 37 °C for 4 h, respectively. CCK-8 solution was added to each well at 37 °C for 1 h, and absorptions of 450 nm were detected. Double-distilled water was used as a negative control. (C) The Vero cells and IPEC-J2 cells were treated with different concentrations of nystatin at 37 °C for 4 h, respectively. CCK-8 solution was added to each well at 37 °C for 1 h, and absorptions of 450 nm were detected. DMSO was used as a negative control. (D) The Vero cells and IPEC-J2 cells were transfected with siCav, and the second transfection was carried out at 24 h after the first transfection. The inference efficiency was detected by qRT-PCR and Western blotting at 24 h after the second transfection. Ctrl means control. (E) The Vero cells (up) and IPEC-J2 cells (down) were treated with MβCD at 37 °C for 1 h, and the amount of cholesterol was detected following the instructions of the cholesterol quantitative kit (AmyJet Scientific). A histogram was created via the fluorescence density. The error bars represent the SD of 10 figures from three independent experiments. *P < 0.05; **0.05 < P < 0.01; ***0.01 < P < 0.001; ****P < 0.001. [file 13567_2020_739_MOESM5_ESM.tif]

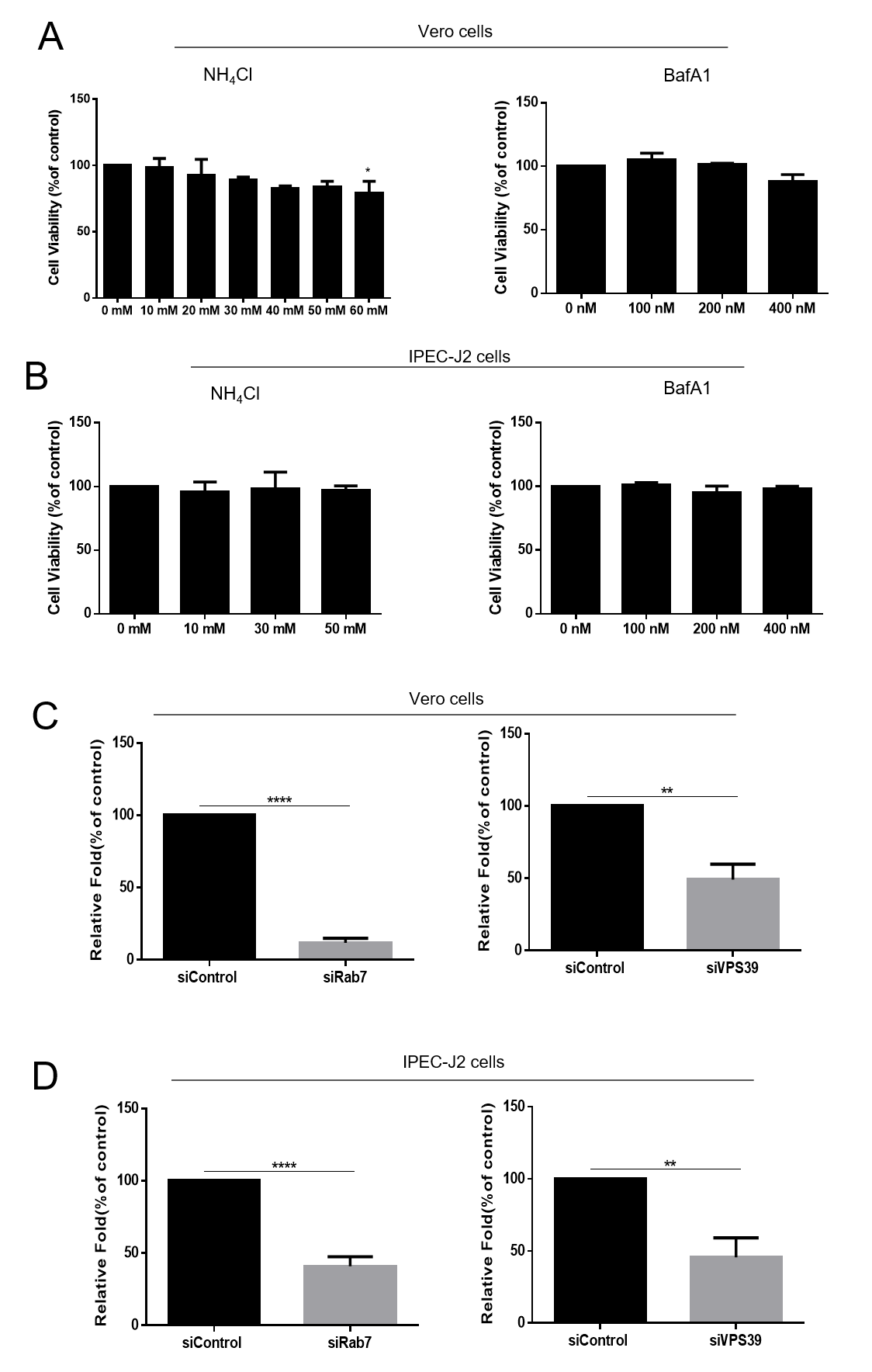

Supplement: Supplementary file 6 — Additional file 6. Internalized PEDV traffics to lysosomes via endosomes. (A, B) Vero cells (A) and IPEC-J2 cells (B) were treated with different concentrations of NH4Cl and Baf A1 at 37 °C for 4 h, respectively. CCK-8 solution was added to each well at 37 °C for 1 h, and absorptions of 450 nm were detected. Double-distilled water and DMSO were used as negative controls, respectively. (C-D) The Vero cells (C) and IPEC-J2 cells (D) were transfected with siRab7 and siVPS39, respectively, and the second transfection was carried out at 24 h after the first transfection. The inference efficiency was detected by qRT-PCR at 24 h after the second transfection. **0.05 < P < 0.01; ***0.01 < P < 0.001; ****P < 0.001. [file 13567_2020_739_MOESM6_ESM.tif]
